# “Seeing inside out”: revealing the effectiveness of otoscopy training in virtual reality enhanced practical exams - a randomized controlled trial

**DOI:** 10.1186/s12909-024-05385-3

**Published:** 2024-04-22

**Authors:** Tobias Albrecht, Nathalie Fehre, Wolf Ramackers, Christoph Nikendei, Christian Offergeld

**Affiliations:** 1 Department of Otorhinolaryngology, Head and Neck Surgery, Medical Center - University of Tuebingen, Tuebingen, Germany; 2 Department of Otorhinolaryngology, Head and Neck Surgery, Medical Center - University of Freiburg, Freiburg, Germany; 3 Department of General, Visceral and Transplant Surgery, Hannover Medical School, Hannover, Germany; 4 Department for General Internal Medicine and Psychosomatics, Medical Center - University of Heidelberg, Heidelberg, Germany

**Keywords:** Medical education, Otoscopy, Virtual reality simulator

## Abstract

**Background:**

The study aimed to assess the impact of different training modalities on otoscopy performance during a practical exam using a high-fidelity simulator and to determine if objective evaluation of otoscopy is feasible using a simulator that records insertion depth and tympanic membrane coverage.

**Methods:**

Participants were assigned to one of four groups: control and three intervention groups with varying training approaches. Participants received otoscopy training and then were assessed through a practical exam on a high-fidelity simulator that uses virtual reality to visualize the ear canal and middle ear. Performance was evaluated using a modified Objective Structured Assessment of Technical Skills checklist and Integrated Procedural Performance Instrument checklist. Insertion depth, tympanic membrane coverage, and correct diagnosis were recorded. Data were tested for normal distribution using the Shapiro-Wilk test. One-way ANOVA and, for non-normally distributed data, Kruskal-Wallis test combined with Dunn’s test for multiple comparisons were used. Interrater reliability was assessed using Cohen’s κ and Intraclass correlation coefficient.

**Results:**

All groups rated their training sessions positively. Performance on the OSATS checklist was similar among groups. IPPI scores indicated comparable patient handling skills. The feedback group examined larger tympanic membrane areas and had higher rates of correct diagnosis. The correct insertion depth was rarely achieved by all participants. Interrater reliability for OSATS was strong. IPPI reliability showed good correlation.

**Conclusion:**

Regardless of training modality, participants perceived learning improvement and skill acquisition. Feedback improved examination performance, indicating simulator-guided training enhances skills. High-fidelity simulator usage in exams provides an objective assessment of performance.

## Background

Otoscopy is used to diagnose a wide range of ear canal and middle ear diseases. Accurate performance of this procedure is vital for valid diagnoses of otologic diseases, which are common in all age groups and can significantly affect the function of this important sensory organ [1]. Otitis media is one of the most common childhood infections, the leading cause of doctors’ visits by children and the most frequent reason children are prescribed antibiotics or undergo surgery in developed countries [2]. Approximately 25% of primary care complaints are related to otolaryngology, and patients with ear pain often consult a general practitioner or pediatrician. These medical professionals must then examine and evaluate the ear canal and the tympanic membrane [3], making otoscopy a crucial skill not only for otorhinolaryngologists but also for other medical disciplines. However, pediatricians and general practitioners often lack proper training in the clinical assessment of the ear [4–6]. Although otoscopy is a crucial procedure in multiple specialties and conditions treated by otolaryngologists account for up to 17% of adult primary care referrals and 50% of pediatric referrals, many medical school curricula in the U.S. do not require a mandatory otolaryngology rotation [3, 7]. Even though an otorhinolaryngology rotation is obligatory during medical school, training is very limited in medical curricula. In addition to didactic lectures and faculty demonstrations, otoscopic examination techniques are often learned and practiced in skills-labs [8, 9]. In peer-to-peer learning, however, there usually are no pathologic findings. The teacher is unable to check whether the student has inserted the instrument correctly, which parts of the tympanic membrane have been examined, and if the structures indicated have actually been recognized. However, to make a correct diagnosis in otoscopy, the otoscope must be inserted to the correct depth and then, due to the otoscope’s limited field of view, the entire ear canal and tympanic membrane must be scanned systematically.

To compensate for these limitations, the use of simulators has increased steadily in the field of medical education in recent years [10], and various simulators for ear examination are available [11–15]. While most current otoscopy simulators are capable of presenting physiological and pathological findings [16], they do not offer accurate depth assessment of the inserted instrument or real-time feedback capability because many simulators still do not allow the instructor to see exactly what their learners are observing. Feedback plays a key role in the acquisition and optimization of clinical skills [17]. Therefore, otoscopy is still a skill difficult to teach [18] and, moreover, remains difficult to assess objectively in the context of a practical examination. The high-fidelity otoscopy simulator investigated in this study [14] displays physiological and pathological findings utilizing virtual reality technology. The instrument’s tip is tracked in three-dimensional space, and a computer determines the corresponding image based on its position relative to the “patient’s” ear. The Image is then displayed on a monitor built into the otoscope. Realistic renderings of the ear canal, the tympanic membrane and middle ear are possible. A training mode enables the user to selectively hide specific structures, like the eardrum, or focus on various areas and structures within the field of view, while being guided during different instructional sessions. In addition, the trainee receives direct feedback on the insertion depth of the instrument. The tutor can follow exactly what the learner sees during the otoscopy on an external monitor and thus provide direct comments and feedback. In addition the simulator records the percentage of the tympanic membrane examined. This feature could be used in a practical examination to objectively evaluate the performance of the actual procedure.

The goal of this study was to determine whether (1) the fidelity of the training tool used to learn otoscopy has an impact on the ability to accurately conduct otoscopy in a practical exam with a high-fidelity simulator and (2) the objective evaluation of otoscopy in a practical exam can be accomplished with the use of a high-fidelity trainer that records the insertion depth of the otoscope and the percentage of the tympanic membrane examined.

## Methods

### Ethical approval

The study was conducted in accordance with the general terms and conditions and approval of the institutional ethics committee (Ethics committee Freiburg, #21-1646) and was registered in the German Clinical Trials Register on 22/06/2002 under the registration number DRKS00027178. Participation was voluntary and participants were informed of the aims of the study and provided informed consent before participating in the study. Students with previous experience in otoscopy were excluded from the study.

### Sample, study design and assignment to study group

173 medical students attending their otolaryngology rotation were recruited to participate in this prospectively designed, cluster-randomized, controlled trial. Participants were trained in otoscopy over the course of three faculty-led instructional sessions followed by a practical exam.

The study design includes three intervention groups (IG) and one control group (CG).

**Fig. 1:**
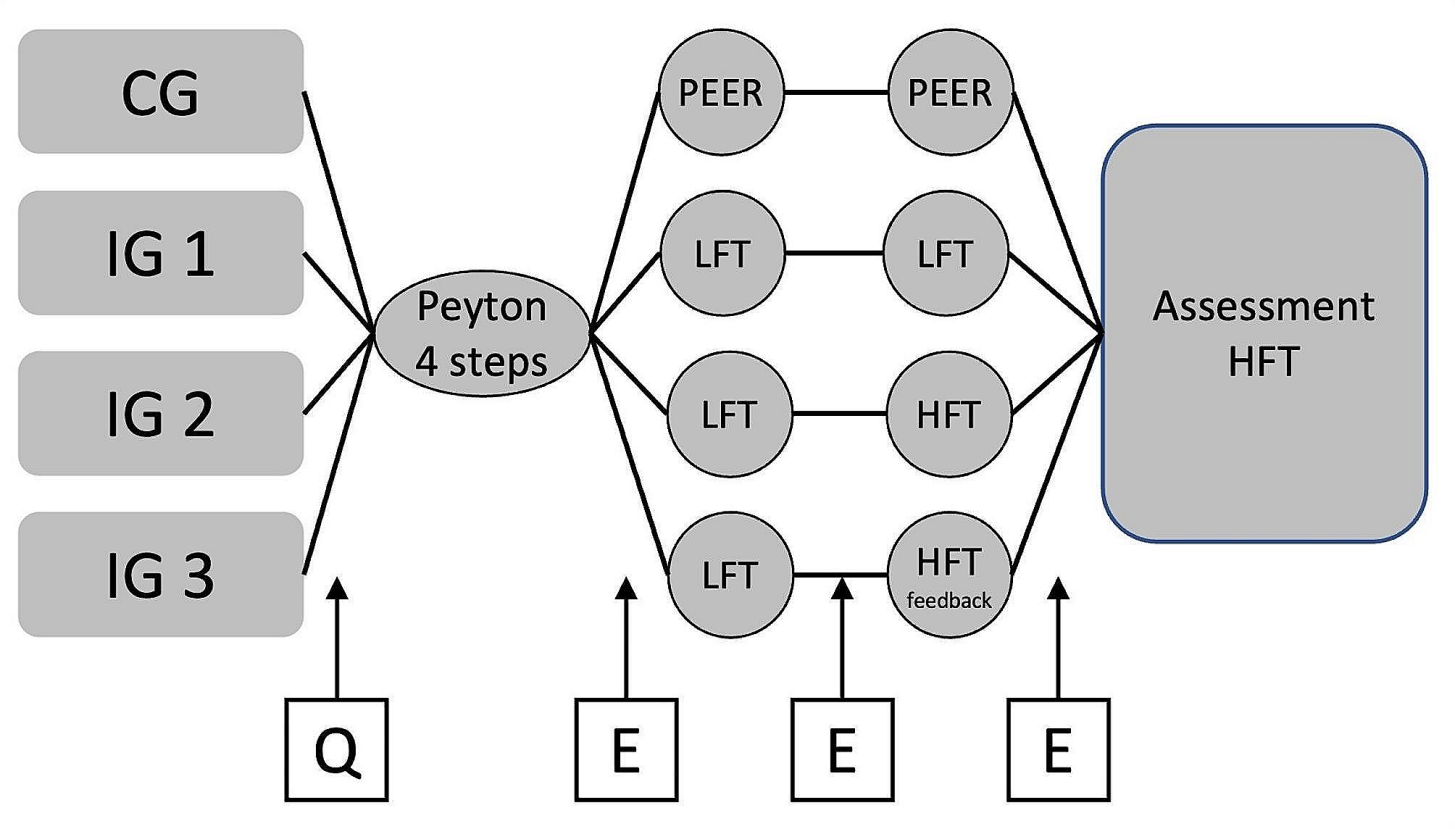
Study design and study course for control group (CG), intervention group 1 (IG1), intervention group 2 (IG2) and intervention group 3 (IG3). LFT = low fidelity trainer; HFT = high fidelity trainer, Questionnaire (Q) collecting baseline data and skill specific self-assessment; Evaluation of the Skill training sessions (E) on a 6-point Likert scale

The first skill training session for all groups was structured according to the Peyton’s 4-step approach [19] and included otoscopy training on a peer. Skill training sessions two and three were conducted with a student to teacher ratio of 1:1 and each participant had five minutes to practice otoscopy. During the instructional sessions, the control group practiced otoscopy only on peers. In intervention group one (IG1), a low-fidelity otoscopy trainer (Blue line, Schultes medacta GmbH, Germany) was used to practice otoscopy during training sessions two and three. The low-fidelity trainer is a model of the right half of a head, featuring a silicone molded pinna and auditory canal. Paper strips with distinct tympanic membrane findings can be inserted through an opening on the side. Hair and the natural curvature of the auditory canal in the transition area from the cartilaginous to the bony part are not depicted, and pathologies in the ear canal cannot be visualized. Intervention group two (IG2) practiced with the low-fidelity trainer in session two and were trained on the high-fidelity trainer (Earsi Otoscope, Haag-Streit Group, Germany) in session three. Intervention group three (IG3) practiced with the low-fidelity simulator during the second instructional session and with the high-fidelity trainer during the third session receiving feedback on insertion depth and percentage of tympanic membrane examined. The high-fidelity simulator was used in the practical exam for all groups (Fig. 1). Physiological and pathological tympanic membrane findings were shown in the intervention groups practicing otoscopy on a model.

### Evaluation prior to training (Q)

To account for any confounding variables, each participant gave information on their age, gender, handedness, past clinical experience relating to clerkships, and qualifications as a paramedic or nurse or previous education in another non-healthcare-related profession, to ensure that conditions were the same in all groups. After an introductory lecture on the otolaryngology examination, participants were asked to self-assess their abilities to perform an ear examination correctly.

### Evaluation of skills training sessions

After every training session the participants were asked to complete an evaluation form to maximize the comparability of the different training sessions. A 6-point Likert scale with 1 = agree completely to 6 = disagree completely was used to evaluate the following seven statements: (1) The course was clearly structured. (2) The contents of the course were conveyed in an understandable manner. (3) Questions and concerns of the students were addressed. (4) My learning improvement is high. (5) The teachers were engaged. (6) The learning atmosphere was pleasant. (7) I was able to acquire practical skills.

### Objective assessment of training

To assess acquired competencies learned during the training sessions, a practical exam was held at the end of the otorhinolaryngology rotation. In the individual exam, participants first had to take an ear-specific medical history with an actor and then examine the ear. Examination was performed on the high-fidelity simulator displaying a pathologic finding matching the anamnesis. Performance of the otoscopy was assessed by two independent raters using a modified Objective Structured Assessment of Technical Skills (OSATS) checklist [14], consisting of the Items (1) the otoscope is held correctly, (2) the otoscope is stabilized, (3) the otoscope is inserted to the correct insertion depth, (4) the auricle is pulled back- and upwards, and (5) the otoscopy is performed atraumatically. In addition, an evaluation of the procedural skills in context was conducted using an Integrated Procedural Performance Instrument (IPPI) checklist [20] by the same two independent raters. The IPPI checklist contained the Items (1) introduced himself/herself to the patient, (2) explained the examination and gave the patient the opportunity to consent, (3) Preparation of the examination, (4) technical performance, (5) being aware of the needs of the patient during examination, (6) professionality (7) overall ability to perform the examination. Time required to examine the tympanic membrane was taken and the percentage of the tympanic membrane examined was recorded. Finally, the participant’s ability to correctly identify the pathological finding was assessed.

### Statistical analysis

An a-priori power analysis with the power of 80% was performed with a total sample size of 175 at a 5% significance level assuming a moderate to strong effect.

The data was tested for normal distribution using the Shapiro-Wilk test. In cases where the data was non-normally distributed, the Kruskal-Wallis test was used to compare the data combined with a Dunn’s test in case of multiple comparison. A one-way ANOVA was used to perform a statistical analysis on the normally distributed data. Statistical significance was defined as a *p*-value less than 0.05. Data are shown as means with standard deviation. Interrater reliability for the OSATS with two raters during the oral-practical exam was assessed using Cohen’s κ, with values between 0.6 and 0.8 considered as strong agreement and > 0.8 as near complete agreement. For the IPPI the intraclass correlation coefficient (ICC) was calculated. Based on the 95% confidence interval of the ICC estimate, values between 0.5 and 0.75, between 0.75 and 0.9, and greater than 0.90 were considered moderate, good, and excellent reliability, respectively [21]. Statistical analyses were performed using SPSS Version 29 (IBM Corp., Armonk, New York; USA).

## Results

### Participants

The baseline values regarding age, gender, semester, handedness, and education in medical profession are shown in Table 1. None of the participants had received prior teaching in otorhinolaryngology.

**Table 1:** Group characteristics of the intervention groups 1–3 (IG 1–3) and the control group (CG)

Characteristics	IG 1 (*n* = 51)	IG 2 (*n* = 48)	IG 3 (*n* = 49)	CG (*n* = 25)
Age; mean (SD)	23.51 (2.49)	25.6 (3.79)	24.06 (3.05)	23.68 (3.06)
Gender; female, N (%)	33 (64.7%)	28 (58.33%)	31 (63.27%)	19 (76%)
Semester; mean (SD)	8 (0.94)	7 (0.84)	7 (0.79)	8 (0.95)
Right-Handedness; N (%)	49 (96.08%)	42 (89.36%)	41 (83.67%)	23 (92%)
Education in medical profession				
-non	39 (76.47%)	28 (58.33%)	32 (65.31%)	19 (76%)
-paramedic	4 (7.84%)	3 (6.25%)	10(20.41%)	3 (12%)
-nurse	2 (3.92%)	10 (20.83%)	5 (10.2%)	1 (4%)
-other (non-healthcare-related)	6 (11.76%)	7 (14.58%)	2 (4.08%)	2 (8%)

### Evaluation of skill training sessions

All training Session were rated on a 6-point Likert scale directly after the session. Overall, the participants rated all training sessions very positively, with a high level of agreement for the different statements describing the courses. The training sessions were rated as clearly structured (≥ 84% agreement). The contents of the course were conveyed in an understandable manner (≥ 91.1% agreement) and questions and concerns of the students were addressed in a pleasant learning atmosphere during all training sessions (≥ 95.3% agreement). With an agreement rate of ≥ 95.8%, all teachers were engaged. Regardless to their group allocation participants rated their learning improvement of each training session as high (≥ 83.3% agreement) and were able to acquire practical skills (≥ 78.3% agreement). Mean values of the results of all three evaluations of the individual groups are shown in Table 2

**Table 2:** Evaluation of all training sessions. Data presented as mean values (SD) of the three intervention groups (IG1-3) and the control group (CG).

Item	IG 1 (*n* = 51)	IG 2 (*n* = 48)	IG 3 (*n* = 49)	CG (*n* = 25)
Clearly structured	1.37 (0.73)	1.54 (0.94)	1.44 (0.86)	1.42 (0.64)
Contents presented in understandable manner	1.33 (0.59)	1.65 (0.98)	1.56 (0.86)	1.38 (57)
Questions / concerns addressed	1.27 (0.57)	1.65 (1.06)	1.6 (0.93)	1.23 (0.43)
Learning improvement high	1.51 (0.65)	1.83 (1.02)	1.74 (0.96)	1.42 (0.64)
Engaged teachers	1.29 (0.61)	1.40 (0.82)	1.34 (0.80)	1.12 (0.33)
Pleasant learning atmosphere	1.31 (0.58)	1.50 (1.11)	1.42 (0.97)	1.27 (0.60)
Acquire practical skills	1.35 (0.56)	1.56 (0.99)	1.52 (0.84)	1.27 (0.99)

### Objective assessment of training

#### OSATS ratings

**Fig. 2:**
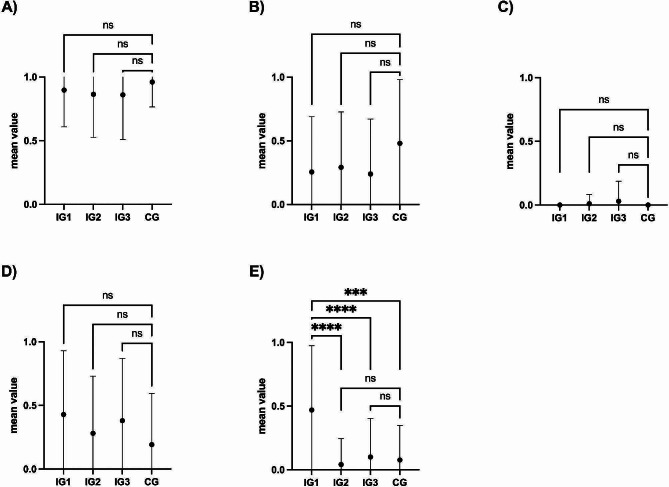
Mean values with standard deviation of the individual items of the modified OSATS checklist. Intervention group 1–3 (IG1-3) and control group (CG); (**A**) Item: the Otoscope is inserted to the correct insertion depth; (**B**) Item: the otoscope is stabilized; (**C**) Item: the auricle is pulled back- & upwards; (**D**) Item: Otoscopy is performed atraumatically; (**E**) Item: The otoscope is held correctly. **** *p* < 0.0001; *** *p* < 0.001

Performance of the otoscopy was assessed using a modified OSATS checklist. Despite the insertion depth, the otoscopy procedure on the high-fidelity model was performed very well and comparably by all groups, regardless of their previous otoscopy training. Only the item “otoscope is held correctly“ showed a significant difference between IG1 and the other groups, where there were no significant differences between the other groups and the other Items. The correct insertion depth was rarely achieved in all groups and was often considered to be too shallow. Mean values of the individual items are shown in Fig. 2

#### IPPI ratings

**Fig. 3:**
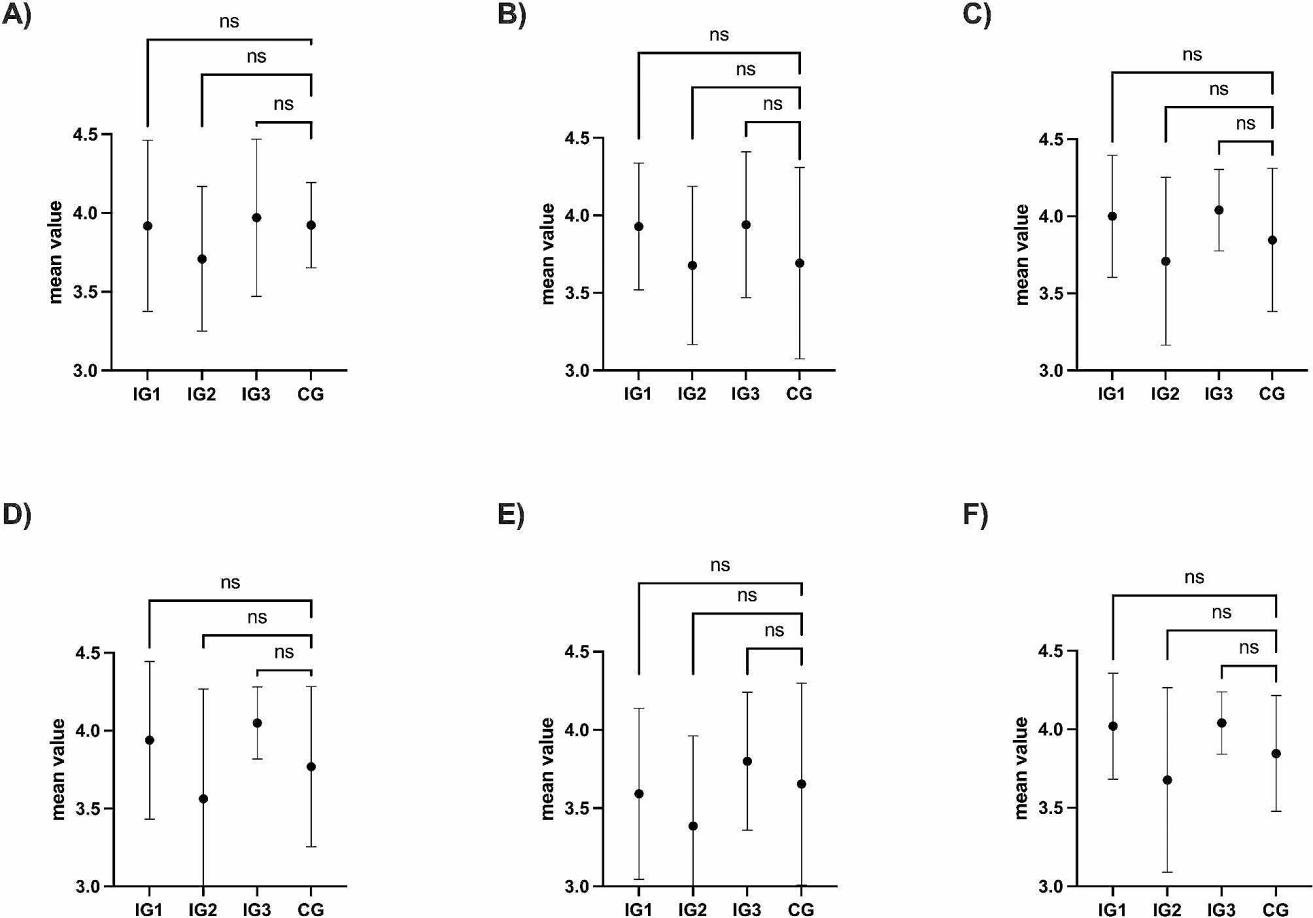
The graphs show the mean and standard deviation of each item on the IPPI checklist. The median and [IQR] of each group are given in parentheses according to the group order shown in the graphs. Intervention group 1–3 (IG1-3) and control group (CG); ns = not significant; (**A**) Item: introduced himself/herself to the patient (4.0 [0]; 4.0 [1]; 4.0 [0]; 4.0 [0]); (**B**) Item: explained the examination and gave the patient the opportunity to consent (4.0 [0]; 4.0 [1]; 4.0 [0]; 4.0 [1]); (**C**) Item: Preparation of the examination (4.0 [0]; 4.0 [1]; 4.0 [0]; 4.0 [0]); (**D**) Item: technical performance (4.0 [0]; 4.0 [1]; 4.0 [0]; 4.0 [0]); (**E**) Item: being aware of the needs of the patient during examination (3.5 [0.5]; 3.5 [1]; 4.0 [0.5]; 4.0 [0.5]); (**F**) Item: professionality (4.0 [0]; 4.0 [1]; 4.0 [0]; 4.0 [0])

The procedural skill in context of the doctor-patient setting during the oral-practical examination was assessed using an IPPI instrument. The items of the IPPI concentrated on the sub-category of communication skills during the procedure, as proposed previously [22], as the technical skills were already covered by the OSATS checklist. All four groups achieved similar scores in the individual Items with no significant differences in “introduced himself/herself to the patient” (IG1: 3.9 (0.5);IG2: 3.6 (0.5); IG3: 3.9 (0.5); CG: 3.8 (0.3), “explained the examination and gave the patient the opportunity to consent” (IG1: 3.8 (0.5); IG2: 3.5 (0.6); IG3: 3.8 (0.5) CG: 3.4 (0.6), “Preparation of the examination”(IG1: 3.9 (0.4); IG2: 3,5(0.6); IG3: 4,0(0.3); CG: 3,6 (0.5), “technical performance” (IG1: 3.8 (0.5); IG2: 3.4 (0.7); IG3: 4.0 (0.2); CG: 3.4 (0.4), “being aware of the needs of the patient during examination” (IG1: 3.6 (0.6); IG2: 3.4 (0.6); IG3: 3.8 (0.4); CG: 3.7 (0.6) and “professionality” (IG1: 3.9 (0.3); IG2: 3.5 (0.1); IG3: 4.0 (0.2); CG: 3.5 (0.3), as shown in Fig. 3

Using the Item “overall ability to perform the examination” as a measure for clinical validation, the absolute number of “competent students” (students who received ratings of “1” and “2”), “borderline students” (ratings “3” and “4”), and “incompetent students” (“5” and “6”) was calculated. 148 students were rated as competent, 25 as borderline and 0 as incompetent with no significant differences among groups

#### Interrater reliability

Performance during the oral-practical exam regarding the procedural skill in context and the practical skill were rated by two independent raters. The interrater reliability for the checklists were very good with a strong or nearly total agreement for the items of the modified OSATS checklist. The individual Cohen’s κ values for the items of the modified OSATS checklist are shown in Table 3. The ICC was calculated for the cumulative IPPI ratings and showed good correlation with an intraclass correlation of 0.867 with a 95% confidence interval between 0.821 and 0.902

**Table 3:** Interrater reliability for the individual items of the modified OSATS checklist assessed with Cohen’s κ

	CG	IG 1	IG 2	IG 3
The otoscope…				
is held correctly	1	1	1	1
is stabilized	0.95	0.95	0.8	1
is inserted to the correct insertion depth	0.78	0.78	0.91	1
Auricle is pulled back- & upwards	1	1	0.66	0.66
Otoscopy is performed atraumatically	1	1	0.95	1

#### Examination time and percentage of eardrum assessed

During the practical exam, time required to examine the tympanic membrane and percentage of the tympanic membrane examined were recorded with the examination time representing the time between insertion and removal of the otoscope

**Fig. 4:**
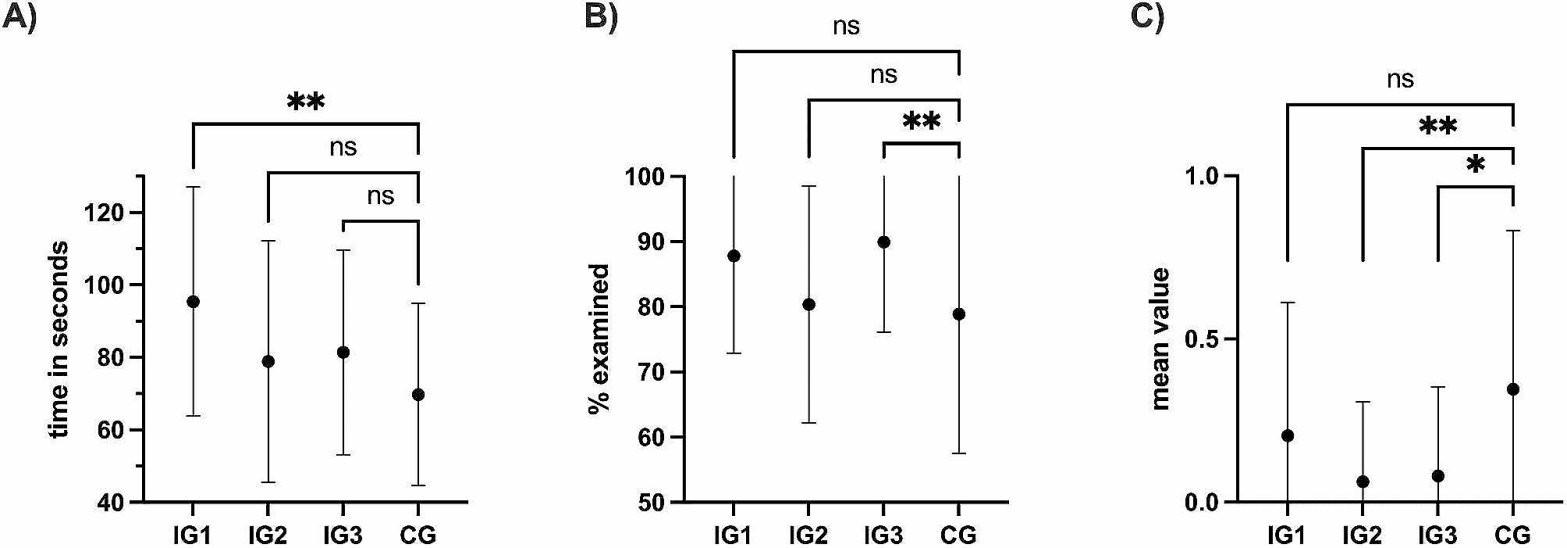
Mean values with standard deviation. Intervention group 1–3 (IG1-3) and control group (CG); ns = not significant; **p < 0.01 * *p* < 0.05  (**A**) time required to examine the tympanic membrane; (**B**) percentage of the tympanic membrane examined; (**C**) provided the correct diagnosis (0 was assigned for a correct and 1 for an incorrect diagnosis)

The average examination time for all participants was 83 seconds with a minimum examination time of 15 and a maximum of 196 seconds. The mean examination times (in seconds) for the individual groups were IG1 (95 ± 32), IG2 (79 ± 33), IG3 (81 ± 28) and CG (70 ± 25), with a significant longer examination time of IG1 compared to IG2 (*p* = 0.03) and CG (*p* = 0.008), but with no significant difference between the CG and the IG2 and IG3 as presented in Fig. 4a

The percentage of the examined tympanic membrane was on average 85% with group differences of 88 ± 15% for IG1, 80 ± 18% for IG2, 90 ± 14% for IG3 and 79 ± 21% in the control group. Significant differences were found comparing CG with IG3 (*p* = 0.007) and IG2 with IG3 (*p* = 0.003) as presented in Fig. 4b

#### Finding the right diagnosis

At the end of the examination, participants were asked about their suspected diagnosis based on the collected medical history and examination findings. 85% of the participants were able to provide the correct diagnosis. Participants of IG2 and IG3 were significantly more likely to provide the correct diagnosis as participants of the CG (*p =* 0.007; *p =* 0.01), with no difference between IG1 and CG as well as between IG1 and IG2/IG3 as shown in Fig. 4c

## Discussion

The present study evaluates the impact of different otoscopy training methods on performance during an exam on a high-fidelity simulator and explores the feasibility of an objective assessment using a high-fidelity trainer that records insertion depth and the percentage of the tympanic membrane examined.

The participating 173 medical students were randomly assigned and shared similar characteristics in terms of their age, gender, semester, handedness, and prior medical education. To ensure a comparable quality of the individual training sessions and thus avoid a potential bias of the performance during the practical examination, the respective training sessions were evaluated by the participants. Participants’ performance on the modified OSATS checklist remained similar across groups. Previous studies have identified otoscopy as an easy-to-learn procedure, with no significant improvement after the third practical teaching session, as measured by instrument handling [18, 23]. In our study, the examination also took place after the third practical lesson. The good results on the OSATS checklist are therefore not surprising and in line with the literature. The significantly poorer performance of IG1 in holding the otoscope could be explained by the fact that this group was trained on the low-fidelity trainer, which only allows examination of the right ear, whereas the high-fidelity trainer simulates examination of the left ear. The correct insertion depth could not be measured in previous studies. As the correct insertion depth of the otoscope is critical for identification of the tympanic membrane, it is interesting to note that it was rarely achieved by the participants and was considered too shallow in 89% of the examinations. Using the IPPI ratings, communication skills during doctor-patient interactions remained consistent across groups, suggesting patient handling was unaffected by training method, which is also reflected in the excellent score in the global item “general ability to perform the examination”, with 85% of the participants being rated competent. Therefore, according to our data, patient handling does not suffer when a skill like otoscopy has been learned on a simulator. Lund et al. were even able to show that teaching of intravenous cannulation skills acquired using simulators in a skills laboratory was superior to bedside teaching when assessed using the IPPI [22]

IG1 took the longest to examine the eardrum, taking an average of 15 seconds longer, without examining a significantly larger area of the tympanic membrane than the other groups. IG2 and IG3 took about the same amount of time, but the feedback group examined the largest part of the eardrum. According to Issenberg, feedback is a key feature of medical simulation that leads to the most effective learning [24]. The higher percentage of tympanic membrane examined could therefore be a result of the feedback IG3 received during their last training session. Although not statistically significant, the highest percentage of correct insertion depths during examination also was achieved by IG3 and possibly more training session with feedback might have yielded a significant difference. The control group took the shortest time for the examination, but also examined the smallest part of the tympanic membrane and was the worst group in determining the correct pathologic finding. Wormald et al. demonstrated in a structured approach to otoscopy training, that the diagnostic ability of study participants tested with photographs of tympanic membranes with chronic otitis media significantly improved after training and concluded, that this teaching approach is likely to be equally beneficial to other otological conditions and to live otoscopy [25]. This statement confirms our observation that IG2 and IG3 were significantly more likely to correctly identify the pathology than the control group, who had never seen a pathological tympanic membrane during their training with peers.

### Limitations

This study has limitations, as do all studies. The IG3 group only received feedback during one training session. Previous studies have demonstrated the benefit of repetitive skills training with feedback in the early acquisition of procedural skills [26]. It is therefore reasonable to assume that repeated feedback may improve performance, but this was not investigated in the present study. Repeated feedback may also lead to an overall improvement in the insertion depth of the otoscope.

The study involved three training-sessions with two 5-minute sessions on a simulator to learn otoscopy. While Polk ML [18] identified otoscopy as an easy-to-learn procedure, additional or longer training sessions may have also resulted in better performance. It is also important to mention that the assessment was completed on a high-fidelity simulator that was only accessible to IG2 and IG3 beforehand. This may have given IG2 and IG3 an advantage in recognizing pathologies, despite having comparable results in the OSATS and the IPPI ratings.

The effect on the ability to accurately perform otoscopy in a hands-on examination with a high-fidelity simulator was assessed by the number of correct items in the OSATS. For this purpose, a power analysis was performed a-prior to the study with a total sample size of 175 participants at a 5% significance level. Assuming a moderate to strong effect size of d = 0.75, the power was 80%. This leaves the study with a 20% probability of a second type error.

## Conclusion

Our study shows that, regardless of the training modality, participants perceived substantial learning improvement and an acquisition of a practical skill. Otoscopy appears to be effectively learned through various teaching methods. The feedback on the high-fidelity simulator notably enhances examination quality in terms of tympanic membrane assessment and accurate diagnosis of pathological findings. The use of a high-fidelity simulator for otoscopy in a practical exam is possible, even without prior training, without compromising the performance. Incorporating a high-fidelity simulator in exams ensures consistent testing conditions and more realism through pathological findings. It allows objective evaluation of performance by measuring the insertion depth and the percentage of the tympanic membrane examined. With the overall poor performance of all groups with respect to insertion depth, this study suggests that otoscopy seems to be a simple examination, the correct procedure may not be as easy to learn as previously assumed by other authors

## Data Availability

All relevant raw data from this study will be available from the corresponding author upon reasonable request for non-commercial purposes.
